# Online screening for excessive daytime sleepiness: a feasibility study

**DOI:** 10.3389/fpsyg.2024.1422555

**Published:** 2024-08-06

**Authors:** Jan Hlodak, Andrea Madarasova Geckova, Simona Carnakovic, Eva Feketeova

**Affiliations:** ^1^Faculty of Social and Economic Sciences, Institute of Applied Psychology, Comenius University, Bratislava, Slovakia; ^2^Institute of Health Psychology and Research Methodology, Medical Faculty, University of Pavol Jozef Safarik, Kosice, Slovakia; ^3^First Department of Psychiatry, Medical Faculty, University of Pavol Jozef Safarik, Kosice, Slovakia; ^4^University Hospital of L. Pasteur, Kosice, Slovakia; ^5^Medical Faculty, Department of Neurology, University of Pavol Jozef Safarik, Kosice, Slovakia

**Keywords:** narcolepsy, online screening, campaign, feasibility, excessive daytime sleepiness

## Abstract

**Purpose:**

Excessive daytime sleepiness (EDS) can have a significant impact on health and quality of life but may remain undiagnosed due to low awareness and underestimation of the clinical impact of the symptoms. An online screening tool supported by media campaigns might increase awareness and help detect undiagnosed cases of EDS and narcolepsy. The aim of this study was to develop an online screening method, along with a media campaign focusing on EDS, and evaluate its feasibility.

**Methods:**

Online screening supported by a media campaign targeting young and middle-aged adults (18–45 years old) were developed and implemented over a period of 1 year starting from November 2022. The Epworth Sleepiness Scale was used to identify EDS, and the Swiss Narcolepsy Scale was used to identify narcolepsy. In addition, the data on sociodemographic characteristics, selected sleep and health indicators and lifestyle behaviors were collected to indicate the etiology of the EDS. Feasibility, e.g., implementation and practicality, was assessed by the response rate, response to the promotion strategy, time spent on the tool, sample characteristics, and the prevalence of identified EDS and narcolepsy cases.

**Results:**

A total of 2,390 people opened the screening link; 568 of them completed the online screening (23.8%), and most of them (*n* = 437, 76.9%) left their contact data to receive feedback. We identified 171 (30.1%) respondents at risk of EDS and 61 (10.7%) at risk of narcolepsy. The mean time of the screening was 15 min.

**Conclusion:**

An online screening tool supported with a campaign seems to be a feasible way to increase awareness about EDS and prevent delayed detection of EDS cases.

## Introduction

EDS is associated with an inability to stay awake during the daytime period, and people with EDS have an urge to fall asleep during day without the possibility of managing their sleep–wake cycle (Brown and Makker, 2020). According to Gandhi et al. (2021), people suffering from EDS mostly complain about feeling an excessive need for sleep, experiencing uncontrollable sleep episodes, prolonged unrefreshing sleeps, repeated daytime naps and the inability to wake up, as well as confusion and automatic behavior.

The gold standard of subjective assessment of EDS is the Epworth Sleepiness Scale (ESS; Johns, 1991), which has been widely used in other EDS screening-like studies. The etiology of EDS could be attributed to various factors, such as Obstructive Sleeping Apnoea (OSA), Circadian Rhythm Sleep Disorders (CRSD), Restless Legs Syndrome (RLS), central disorders of hypersomnolence (narcolepsy, idiopathic hypersomnia, etc.) and psychiatric disorders.

Currently available studies which used screening-like methods for measuring EDS and possible etiologies inspired our research methodology. For example, Schwegler et al. (2006) implement online EDS screening in pharmacy customers in Sweden (*n* = 4,901), focusing on OSA, psychiatric disorders, RLS and Narcolepsy. In their sample, 16.5% of females and 23.9% of males manifested EDS symptomatology, measured using the ESS (Johns, 1991).

Tran et al. (2009) aimed to develop a feasible screening tool for EDS, insomnia, OSA and RLS. From 84 pharmacy customers recruited to fill in the questionnaire, 7.1% of respondents suffer with EDS, 27.4% were at risk of developing RLS, 21.4% scored higher in the screening for OSA, and 33.35% of the sample screened higher for insomnia symptomatology.

Pascoe et al. (2020) delivered an online sleep disorder screening for healthcare workers using the ESS together with tools measuring insomnia, OSA, and Circadian Rhythm Sleep Disorder (CRSD) risks. Among 2,851 healthcare providers, 30.7% of caregivers showed symptoms of EDS, with those working night shifts being in the upper half; 27.5% of participants scored higher in insomnia symptoms in evening or night shift workers, while 36.9% of respondents scored higher for possible OSA symptoms.

Bartlett et al. (2008) approached 10,000 randomly selected Australian citizens by postal mail using a screening battery consisting of tools for measuring sleep quality, with the Pittsburgh Sleep Quality Index (Buysse et al., 1988), the EDS with Epworth Sleepiness Scale (Johns, 1991), and the Athens Insomnia Scale (Soldatos et al., 2003), to measure insomnia **s**ymptoms. From 3,300 respondents, 11.7% scored significantly higher in the ESS scores.

In recent studies, around 15–20% of the general population suffers from EDS (Brown and Makker, 2020; Gandhi et al., 2021). A telephone interview study in the US found excessive daytime sleepiness in over 27% of the more than 15,000 individuals interviewed (Ohayon et al., 2012).

The earliest estimate of the prevalence of narcolepsy in Europe comes from the works of Roth in 1957 (Roth and Broughton, 1980). In Czechoslovakia, he estimated the prevalence of narcolepsy at 0.02 and 0.03%. This estimate also included ‘monosymptomatic’ patients, who were very likely also patients with sleep-disordered breathing, given that the link between sleep apnea and EDS was discovered later.

The Slovak Register of Narcolepsy estimates the prevalence of narcolepsy at 0.0011% (Feketeova et al., 2020). In comparison to published European epidemiological data (Norway 0.022%, Heier et al., 2009; Catalonia, Spain 0.0052%, Tió et al., 2018; Germany 0.018, Kallweit et al., 2022), the prevalence of narcolepsy in Slovakia is significantly lower. One possible reason for the low prevalence rates in Slovakia may be the insufficient referral of patients for somnological examinations. As described by Tran et al. (2009), low detection of sleep disorders in public is probably caused by a poor knowledge and awareness, a lack of resources in diagnostics and healthcare continuity.

Therefore, we developed an online screening tool and various dissemination tools with the aim of raising awareness about EDS disorders and participation in our screening through a media campaign and web survey. In contrast to previous published works, e.g., Schwegler et al. (2006), Tran et al. (2009), Pascoe et al. (2020), and Bartlett et al. (2008), our campaign targeted the general population, focusing on young and middle-aged adults. The presented study aimed to assess the feasibility of a web survey in detecting undiagnosed cases of EDS.

## Methods

### Procedure

#### Development of the screening tool

Based on a literature review on sleep quality and a previous screening carried out in other countries (e.g., Switzerland, Australia, United States) we identified the relevant concepts and measurement tools for screening for EDS and its etiology. These were consulted with somnology, psychiatry and psychology experts to assure that gaps in screening will be covered. Questionnaires that were not available in Slovak language were back translated, and the final questionnaire battery was assessed in a pilot study to ensure its understandability and length.

#### Implementation of screening and dissemination

**Figure 1:**
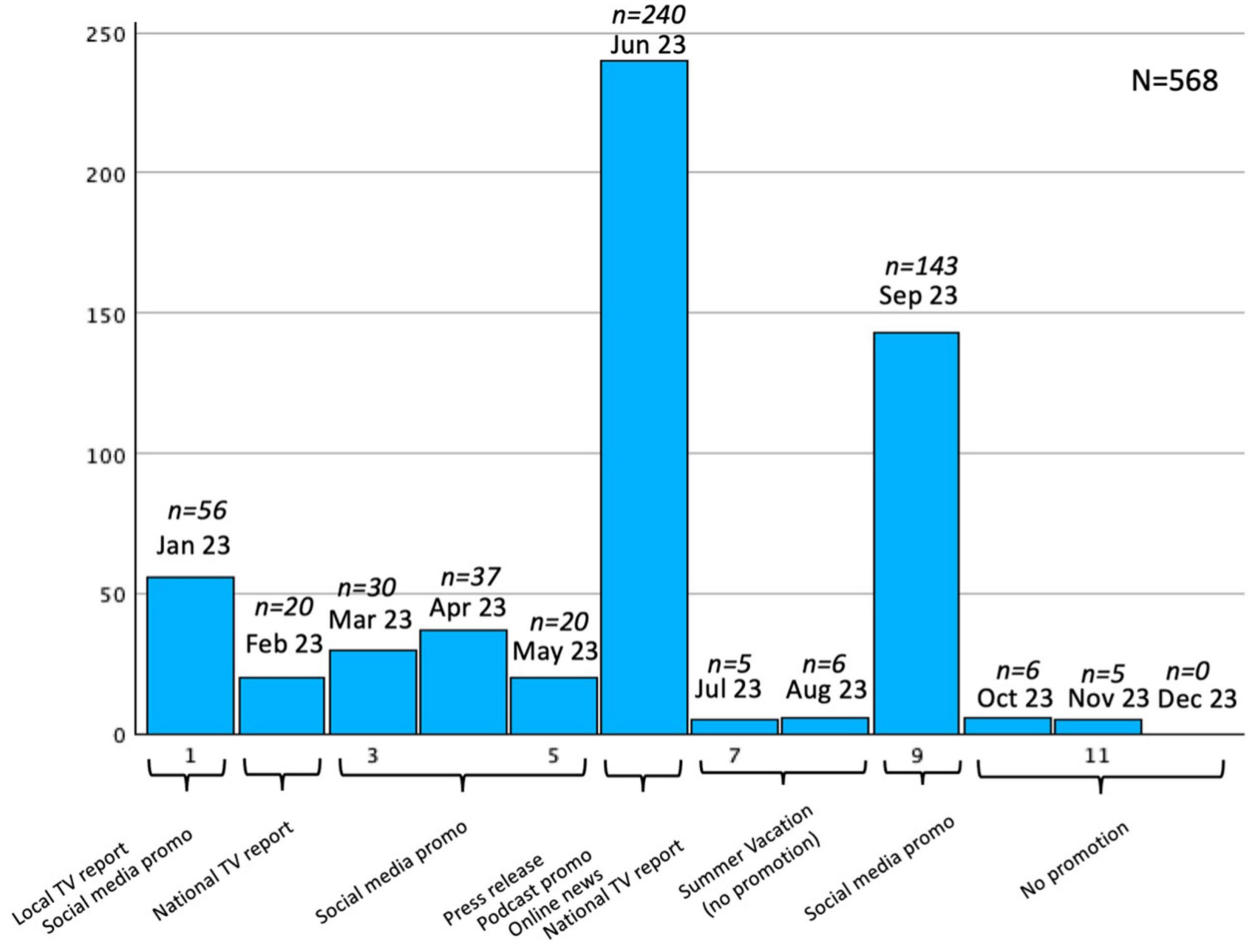
Promotion strategy and its impact on sample increase.

The screening battery was published online on the webpage care4health.sk in November 2022. To approach the widest range of people who possibly suffer from EDS but do not seek medical help, we decided to focus on a traditional media and social media campaign, in which we spread information about EDS and Narcolepsy and introduced our online screening to facilitate people who sleep a lot during the day or tend to fall asleep in undesired situations (e.g., at work, while standing, while talking, etc.) to undergo a screening. Various dissemination tools were used, e.g., a press release, television news reports, social media posts, blogs, podcasts, and printed flyers. These tools were disseminated across the country mainly in first few months of the implementation. For more details, see Figure 1. The link to the online screening was embedded in every dissemination tool. The possibility to get feedback was offered to respondents who left us their e-mail address, and in case of significant EDS and/or narcolepsy scores a participant was given the option to undergo a clinical interview and later a diagnostic process in a sleep laboratory, if necessary.

#### Screening tool

The screening battery was constructed to be the most effective but also as short as possible for people who might tend to fall asleep while filling it in. It consists of the following segments: (1) sociodemographic data, (2) sleep-related indicators, (3) health and health-related quality of life indicators, (4) lifestyle indicators.

#### Sociodemographic indicators

The screening tool begins with questions tracking sociodemographic indicators: age, gender, education (elementary, apprenticeship, secondary with school-leaving examination, university), employment status (student, full-time employed, part-time employed, self-employed, casual worker, unemployed, career, disability pensioner, retired). Perceived socioeconomic status was assessed using the Socioeconomic Status (SES) ladder (Adler et al., 2000) on a 10-point graphic scale (1 = very low SES; 10 = very high SES) and self-assessed item for family affluence measured on a Likert scale: very good, rather good, average, not bad, bad. Respondents were also asked about their living conditions with response options: living with spouse, living with partner, living with family members, e.g., children, parents, living alone.

#### Sleep-related indicators

EDS was measured using the ***Epworth Sleepiness Scale* (*ESS*)** referring to the possibility of napping in eight daily situations on a scale from 0 to 3, where 0 corresponds to none and 3 to a high napping probability (Johns, 1991; Johns, 1992). The sum score varies from 0 to 24, while a score higher than 10 (the cutoff point) identifies individuals most likely to have EDS. This tool was translated form the Czech version of the scale which is most similar to Slovak (Schálek et al., 2015).

The ***Swiss Narcolepsy Scale* (*SNS*)** consists of five questions mapping the severity of selected narcolepsy symptoms. Respondents answer on a 5-point scale from 1 (never) to 5 (almost always). The score is calculated using a specific formula (range from −110 to 66), and a score lower than 0 indicates the presence of narcolepsy (Sturzenegger et al., 2018). The scale was translated for the purpose of this study and tested as a pilot before the screening publication.

The ***Pittsburgh Sleep Quality Index* (*PSQI*)** consists of 19 items mapping sleep quality, grouped in the following 7 areas: subjective sleep quality, sleep latency, duration of sleep, efficiency of sleep, sleep interruptions, use of a sleep aid, tiredness during the day. Each area scale is weighted in a range from 0 (no problems) to 3 (significant problems). Together they result in an index of sleep quality as the sum of all areas (Buysse et al., 1988), with a score higher than 5 points indicating experiences of sleep-related difficulties (Alves et al., 2023). The Slovak translation of this questionnaire was available (Backhaus and Rieman, 2003).

Respondents were asked to fill in three additional questions mapping health care utilization (“Have you ever been diagnosed with a sleep disorder?”), restless legs (“Is your sleep interrupted by unpleasant feelings in your legs which occur only in the night-time or during times of rest?”), and apnea (“Do you snore with breathing pauses?”), and they could answer yes or no.

#### Health and health-related quality of life indicators

For subjective health evaluation we used the ***Health Survey Short Form 12* (*SF-12*)**, which consists of 12 items measuring mental and physical health. The *Mental component score (MSC)* is measured using 5 items in 4 components: vitality, social functioning, role emotional, and mental health. The *Physical component score (PCS)* is measured using 7 items in another 4 components: physical functioning, role physical, bodily pain, and general health, with Likert scale answers options, e.g., *1 excellent, 2 very good, 3 good, 4 fair, 5 poor,* and *yes* or *no* answer option. These answers are then calculated to raw scores and transformed to a summary scale with a mean of 50 points and standard deviation of 10 points and an overall score with a maximum of 100 points. Higher scores indicate better quality of life and mental/physical health (Ware et al., 1995, 1996). The scale was translated for the purpose of this study and tested as a pilot before the screening publication.

We used one item from SF-12 and reformulated it into an additional item asking about the frequency of the impact of sleep on everyday work activities [“*During the past 4 weeks, how much did* sleep *interfere with your normal work (including both work outside the home and housework)?”*] with answer option: *1 not at all, 2 a little bit, 3 moderately, 4 quite a bit, 5 extremely.*

The ***Hospital Anxiety and Depression Scale* (*HADS*)** was used to measure symptoms of depression and anxiety. The questionnaire consists of 14 items, 7 measuring anxiety symptoms and 7 for depression symptoms, and questions are focused on how the respondent felt during the last week. The answer option measure frequency on a Likert-like scale from 0 (most of the time) to 3 (not at all), and the total raw score for each segment can vary from 0 to 21 (Zigmond and Snaith, 1983). The Slovak version of the questionnaire was adapted from the study of Skoumalova et al. (2020). According to the authors, interpretation of this score is categorized as 0–7 (normal), 8–10 (borderline) and 11–21 (abnormal level).

Additional closed question to evaluate physical health of respondents were*: “Do you suffer from chronic diseases?”* with response choices, e.g., headaches, diabetes, oncological, cardiovascular, asthma, thyroid disease, psychiatric disease, other. For closer evaluation of mental health, we added 2 questions: *“Are you under treatment of a psychiatrist?”* and *“Do you use drugs for depression?”* (with yes/no response options). To evaluate body structure and its possible impact on OSA, we used a graphical scale of body types for respondent to choose the one closest to their body-shape, with 9 choices from slim/normal (1–4) to overweight (5–9).

### Lifestyle indicators

Every respondent had to answer the question of whether he or she used to play videogames, with a yes/no answer option. If yes, a screening for a gaming disorder was shown. The ***Internet Gaming Disorder Scale* (*Short Form*)** was used to measure excessive gaming activities, which could result in sleep problems or EDS. The scale consists of 9 questions with yes/no response options. The raw score was calculated by summing the “yes” responses, and a cut-off score ≥ 4 points indicates excessive gaming habits. Two questions in this instrument are additional for qualitative analysis of frequency of playing games in days and time spend playing. These questions are not counted in the raw score (Lemmens et al., 2015).

The ***Excessive Internet Use Scale*** was used for measuring possible interference of Internet behavioral activities and sleep quality. Five questions were used to evaluate Internet-use addiction with yes or no response options. The raw score was calculated by summing all the “yes” options, with a cut-off ≥3 points indicating possible excessive Internet use (Škařupková et al., 2015). An additional closed question asks respondents about the most common online activity, with the answer options: *“Online gaming, Online shopping, Social media and communication with others, Watching online videos,”* and is not included in the raw score. Both scales were adapted from the questionnaire battery of the national Health-behavior of School-aged Children study (Madarasova Geckova and Husarova, 2023).

The last measuring tool was the ***International Physical Activity Questionnaire Short Form 7* (*IPAQ-7*)** used to measure respondents’ physical activities. It is focused on the physical activity of the respondent in a typical week and consists of 7 open questions focused on high, medium, and low physical activities and on the time of daily sitting. A respondent must answer with the number of days and a mean time in hours and minutes a day of performing a specific intensity of activity. A formula for calculating low, moderate, and high levels of overall physical activity is used to measure each respondent (Hagströmer et al., 2006). The scale was translated for the purpose of this study and tested as a pilot before the screening publication.

Additional questions used to evaluate caffeine drinking habits were focused on coffee and energy drinks consumption (*“Do you drink coffee/energy drink at least once a day?”*) with a yes/no answer options, to evaluate possible sleep disturbance cause by high caffeine consumption during the day.

### Statistical analysis

Feasibility was assessed using descriptive statistics according to Bowen et al. (2009) with a focus on implementation with response rate (number of people approaching link/filling in the tool); response to the promotion strategy and its practicality (time spent on tool, sample characteristics, prevalence of identified EDS and narcolepsy cases), and if we are able to identify EDS and narcolepsy cases using this screening method.

## Results

### Number of people approaching the link and filling in the screening tool

From January to December 2023, a total of 2,390 people opened the screening link (online or via scanned QR codes), and 568 of them completed the online screening, which is a 23.8% response rate. Most of them (437 respondents, 76.9%) left their email addresses to receive feedback from the research team about their results and recommendations.

### Response on promotion strategy

The dissemination plan described above was used to spread information about EDS symptomatology and this online screening. Figure 1 provides a closer look at the time of dissemination strategies realization and its impact.

As shown above, the biggest sample increase happened in June and September. The most effective way of catching the right audience was most likely a press release, which generated 10 of online news articles, which led reader to a webpage link for our online survey. Second, a well-known Slovakian podcast promoted our research in one of their episodes while talking about Narcolepsy topics. The social media campaign used in September was more effective than before, because more people were involved in the online information dissemination and research promotion. Without any promotion (in summer and at the end of the year) the sample increased only slightly. Television reports on local and national television stations were not as impactful as other promotion possibilities. The majority of our respondents (*n* = 141, 24.82%) found the link for this screening from online news reports, which published online news article based on the press release. The social media campaign steered at least one-fourth of respondents on Instagram (*n* = 72; 12.67%) or Facebook (*n* = 92; 16.19%). Using social media campaign and paper-like posters with a link to care4health.sk webpage generated 55 respondents (9.68%). Four respondents (0.70%) found this screening on other platforms, e.g., someone sent them by e-mail, or they searched for it on Google. The questionnaire platform was unable to record this information in 204 (35.91%) missing cases.

### Time spent on the tool

The shortest time to fill in the online tool was 6 min and the longest was more than hour (64 min). The mean time of answering was around 17 min (mean = 17.7, st. dev. = 8.66), with the median around 15 min.

### Sample characteristics

The sample consisted of 68.3% females, 26.9% males, while 27 (4.8%) respondents did not state their sex. The age of the respondents ranged from 16 to 80 years, with the mean age around 34 years, median age 31 years, and modus 21 years. Half of them lived with family members (*n* = 290, 51.1%). The majority of people had finished college education (*n* = 292, 51.4%) and secondary education with school-leaving examination (*n* = 230, 40.5%). Nearly half of respondents were employed full-time (*n* = 271, 47.7%), and one-third of them were (also) students (*n* = 184, 32.4%). Two-thirds of respondents perceive their family affluence as rather good or good (62.8%). More details on the socioeconomic and other demographic data are presented in Table 1.

**Table 1:** Sociodemographic characteristics of the sample (*n* = 568, Slovakia, Jan.–Dec. 2023).

		Prevalence n (in%)	Min-Max Mean (std.dev.)	Missing variables n (in%)
Gender	Male	153 (26.9)		27 (4.8)
	Female	388 (68.3)		
Age			16–80 34.14 (12.90)	6 (1.1)
Living conditions	Living with a spouse	78 (13.7)		2 (0.4)
	Living with a partner	72 (12.7)		
	Living with family members	290 (51.1)		
	Living alone	78 (13.7)		
	Other	48 (8.5)		
Education	Elementary	11 (1.9)		6 (1.1)
	Without school-leaving exam.	29 (5.1)		
	With school-leaving exam.	230 (40.5)		
	University	292 (51.4)		
Employment status^1^	Student	184 (32.4)		
	Full-time employed	271 (47.7)		
	Part-time employed	58 (10.2)		
	Self-employed	55 (9.7)		
	Casual worker	35 (6.2)		
	Unemployed	14 (2.5)		
	Carer	23 (4.0)		
	Disability pensioner	13 (2.3)		
	Retired	14 (2.5)		
Perceived family affluence	Very good	59 (10.4)		5 (0.9)
	Rather good	241 (42.4)		
	Average	205 (36.1)		
	Not bad	53 (9.3)		
	Bad	5 (0.9)		
Perceived socioeconomic status			0–10 6.27 (1.64)	5 (0.9)
Nocturnal shift work		53 (9.3)		7 (1.2)

1 Respondents could mark more options.

### Indicators of sleep quality and quantity

Out of these respondents we identified 171 (30.1%) respondents at risk of EDS and 61 (10.7%) at risk of narcolepsy. The most negative impact (highest mean score) is shown in sleep quality, sleep latency, sleep interruptions and tiredness during the day. The smallest impact on the overall sleep index is provided mostly by the necessity of using sleep aid, sleep efficiency and sleep duration. According to PSQI data, the mean time spent in bed was 7.3 h (mode = 8.0, median = 8.0), and the mean of effective sleep in our sample was 5.4 h (mode = 6.0, median = 6.0). Moreover, 5.1% respondents reported being diagnosed with a sleep disorder, and 22.2% stated the occurrence of sleep disruptions caused by unpleasant feelings in the legs during the night and 7.7% by snoring. More details on the sleep-related indicators of the respondents are provided in Table 2.

**Table 2:** Sleep-related indicators.

		Prevalence n (in%)	Min-Max Mean (std.dev.)	Missing variables n (in%)
Excessive daytime sleepiness (ESS)	Yes (score > 10)	171 (30.1)	0–23 8.60 (4.29)	1 (0.2)
	No	396 (69.7)		
Narcolepsy (SNS)	Yes (score < 0)	61 (10.7)	−48–66 23.34 (19.57)	2 (0.4)
	No	505 (88.9)		
Sleep quality (PSQI)	Sleep quality		0–3 1.48 (0.74)	2 (0.4)
	Sleep latency		0–3 1.39 (1.06)	6 (1.1)
	Sleep duration		0–3 0.93 (1.04)	5 (0.9)
	Sleep efficiency		0–3 0.84 (1.08)	7 (1.2)
	Sleep interruptions		0–3 1.34 (0.60)	19 (0.0)
	Use of sleeping aid		0–3 0.33 (0.85)	0 (0.0)
	Tiredness during a day		0–3 1.33 (0.73)	1 (0.0)
	Total score		1–20 7.60 (3.89)	31 (5.5)
PSQI >5	Yes		344 (60.5%)	31 (5.5)
	No		193 (34%)	
Been diagnosed with a sleep disorder	Yes	29 (5.1)		8 (1.4)
	No	531 (95.5)		
Sleep interrupted by unpleasant feelings in the legs during the night	Yes	126 (22.2)		1 (0.2)
	No	358 (63.0)		
	I do not know	83 (14.6)		
Snore with breathing pauses	Yes	44 (7.7)		0 (0.0)
	No	330 (58.1)		
	I do not know	194 (34.2)		

### Health-related quality of life

The overall quality of life (QoL) of our respondents was measured using the SF-12 with MSC and PSC indicators showing various ranges of QoL indicators, mostly better in the physical scale by a few points (see Table 3). The mean of the overall QoL is 83.03 points out of 100 max. These scores could be affected by the high scores on the anxiety scale (occurring in 31.7% of our sample) and depression (occurring in 13.4% of the sample). The most common diseases that could affect sleep quality and quantity were headaches (19%), psychiatric disorders (8.6%) and Thyroid disease (6.5%). Respondents reported a wide range of other diseases in the open-ended question, mostly allergies, (chronic) pain and high blood pressure. A total of 57 (10%) respondents were also medicated with antidepressants, and 59 (10.4%) visited a psychiatrist. According to the graphic scale and subjective ratings of respondents’ body shapes, most (85.7%) of our respondents are not overweight or obese.

**Table 3:** Health and health-related quality of life indicators.

		Prevalence n (in%)	Min-Max Mean (std.dev.)	Missing variables n (in%)
Quality of Life (SF-12)	Overall SF-12 score		59.28–99.68 83.03 (8.83)	7 (1.2)
	MSC-12 PSC-12		21.57–55.91 39.77 (7.72) 21.62–61.61 43.26 (5.86)	7 (1.2) 7 (1.2)
Anxiety (HADS)	Low	254 (44.7)	0–21	3 (0.5)	Moderate	131 (23.1)	8.28 (4.55)		High	180 (31.7)			Depression (HADS)
	Low	387 (68.1)	0–18	2 (0.4)
	Moderate	103 (18.1)	5.70 (3.96)	
	High	76 (13.4)		
Chronic diseases	Headaches	108 (19.0)		
	Diabetes	12 (2.1)		
	Oncological disease	3 (0.5)		
	Cardiovascular disease	31 (5.5) 28 (4.9) 37 (6.5) 49 (8.6) 139 (24.47)			Asthma	Thyroid disease	Psychiatric disease	Other^1^
Psychiatric patient	Visits psychiatrists	59 (10.4)		5 (0.9)
	Medicated w/ antidepressants	57 (10.0)		5 (0.9)
Body shape	Considered overweight	81 (14.3)	1–9 2.88 (1.55)	0 (0.0)

1 E.g., allergies (*n* = 19), pain (*n* = 15), high blood pressure (*n* = 10), inflammation (*n* = 3), celiac disease (*n* = 3), other (*n* = 89).

### Evaluation of lifestyle indicators

Respondents showed the possibility of excessive gaming bordering with addiction in 4.9% of cases. Most respondents did not play videogames at all (85%). The mean number of days spent playing videogames was 2–3 days a week, with an average time of 2–4 h a day. Internet use was found to border addiction level in 29.4% of respondents. The most common online activities in our sample were communicating and social media use and watching online videos.

The majority of the respondents (39.3%) showed high levels of physical activity during a 7-day retrospective description of hours and minutes spent doing intense, moderate, and low physical activities. The mean time of intense exercise (e.g., gym, fitness, etc.) in our sample was 60.3 min a day; the mean time of moderate activity (e.g., cycling, playing tennis, etc.) was 46.0 min a day; the mean time of low physical activity (e.g., walking) was 115.8 min a day, and the mean time of sitting was 5.5 h a day.

The majority of respondents drank at least one cup of coffee a day (71.7%), but only a few (6%) drank energy drinks at least once a day. Twenty-seven respondents (4.8%) drank both coffee and energy drink at least once a day. For more specific information, see Table 4.

**Table 4:** Lifestyle indicators.

		Prevalence n (in%)	Min-Max Mean (std.dev.)	Missing variables n (in%)
Excessive Gaming	Yes (≥ 4) No Do not play games	28 (4.9) 57 (10.0) 483 (85.0)	0–7 1.54 (1.66)	
	Number of days playing		1–5 3.18 (1.55)	46 (8.09)
	Time of playing		1–5 1.68 (0.8)	46 (8.09)
Excessive Internet Use	Yes (≥ 3) No	167 (29.4) 263 (46.3)	0–5 1.71 (1.47)	138 (24.3)
Reasons of Internet Use^1^	Online gaming Online shopping Social media and communication Watching videos	36 (6.3) 56 (9.9) 379 (66.7) 256 (45.1)		
Physical Activity (IPAQ-7)	Low	82 (14.4)	0–6.14^2^	78 (13.7)
	Moderate	185 (32.6)	3.2 (3.65)	
	High	223 (39.3)		
Coffee drinks use	Yes	407 (71.7)		2 (0.4)
	No	159 (28.0)		
Energy drinks use	Yes	34 (6.0)		5 (0.9)
	No	529 (93.1)		

^1^Respondent could mark multiple options, ^2^Hours per day in last 7 days.

## Discussion

This study aimed to develop an online screening method along with a media campaign focusing on EDS and evaluate its feasibility, e.g., its implementation and practicality in detecting undiagnosed cases of EDS and narcolepsy.

First of all, we wanted to know to what extent this screening tool could be delivered to the intended participants. Our findings indicate sufficient uptake, e.g., 2,390 people opened the screening link, 568 respondents completed it, and 76.9% left their contact data to receive feedback, which enabled an invitation for diagnostics. Implementation of this tool enabled us to identify 171 respondents at risk of EDS (30.1%) and 61 at risk of narcolepsy (10.7%). This number is comparable with the percentage of EDS-positive respondents (30.7%) in the study of Pascoe et al. (2020) and 40.4% of EDS positive respondents in the sample of Schwegler et al. (2006). On the other hand, Bartlett et al. (2008) found only 11.7% of a general population sample to be positive for EDS, and Tran et al. (2009) found only 7.1%. In comparison with these studies, we focused on young and middle-aged adults in the general Slovak population as a first screening method study focused on sleep quality, EDS, and its possible etiology. The screening was published and disseminated online, not only available for pharmacy customers (like Schwegler et al., 2006; Tran et al., 2009) or strictly focused on a certain population group (like Pascoe et al., 2020) based on one characteristic. Bartlett et al. (2008), for example, did focus on the general population but distributed their screening using a physical mailing strategy.

Second, we wanted to learn more about the ability of participants to respond to the tool. Most of the respondents spent around 15 min filling in the screening tool, which is a reasonable time for intended participants to invest in the screening tool.

From previous studies (Schwegler et al., 2006; Bartlett et al., 2008; Tran et al., 2009; Pascoe et al., 2020) we learned that online screening interventions seem to be a promising method enabling feasible and accessible screening for EDS, including its etiology (OSA, RLS, CRSD, central disorders of hypersomnolence, insomnia, or mental health). However, these findings are based on only a few studies implemented on very specific groups with limited dissemination. Our study supports these findings focusing on young and middle-aged adults and added new insight about the implementation of social and mass media campaigns. The whole campaign was focused on young adults and middle-aged adults; therefore, we mainly focused on the online space. In our study, six strategies of dissemination were used. A podcast and a press release, which generated most of our sample in June 2023 and social media campaign in September 2023, were shown to be the most efficient. The sample increase after campaign exposure is supported by other similar research articles findings, e.g., Durkin et al. (2019), who increased bowel cancer screening participation after a media campaign; Morrell et al. (2009), who increased their cervical cancer screening sample with television advertising; and Hersberger et al. (2006), who delivered a feasible online screening for sleep disorders in pharmacies using posters and flyers. Therefore, the data on our sample increase and decrease are most likely connected to the feasibility of our mass and social media campaigns.

We believe the strengths of our screening tool are mostly the relatively short length but also relatively good informativeness for measuring EDS and its possible etiology. Therefore, according to these indicators and the number of EDS-positive respondents described above, we believe our methodological approach of online screening is feasible and could be beneficial for general practices and specialists for rapid screening of EDS and other indicators. On the other hand, online screening methods could be beneficial for those who believe they suffer from EDS and seek non-medical help. Even more in the case of rare hypersomnias, such as narcolepsy or idiopathic hypersomnia, patients with mild or moderate severity of the disorders could be overlooked if they are not referred to a somnologist.

The limitations of our screening method are first, the time-consuming instruction and informed consent approval, which took some time for the respondents to read and could discourage possible respondents; therefore, it was shortened after approximately a month. The screening was also constructed to be sufficiently divided and contained a progress bar to maximize the motivation to fill out the questionnaire. Second, the whole screening tool could be shorter by considering skipping the lifestyle indicator, which is currently determined by the largest amount of missing data in the Excessive Internet Use Scale (Škařupková et al., 2015) and IPAQ-7 (Hagströmer et al., 2006). Also, some of the questionnaires were not clinically validated before the start of this study. Third, we did not directly ask the respondents to state where they found out about our screening (or which campaign strategy aimed them specifically), and we collected these data according to the date of answers being submitted and compared them with the dates of the campaign triggers. Fourth, we did not focus on measuring possible insomnia, which could direct in EDS with a specific questionnaire, as in comparison with, e.g., Tran et al. (2009). Therefore, clinical interviews with those who scored higher in ESS is necessary to identify the accuracy and etiology of EDS by a specialist and compare it with the results of our findings. This stage of research is currently in the process of being carried out. For future directions we recommend a continued mass media campaign, with a bigger number of researchers triggering social media. With the possibilities of funding, greater promotion strategies could be done in comparison with our non-funded research project, leading to a larger sample size and better chances of etiological determination of respondents with possible EDS.

## Conclusion

This study focused on the possibilities of an online screening questionnaire tool aimed at people with excessive daytime sleepiness and those who want to understand more about their sleep quality and quantity and other indicators, such as health-related quality of life and lifestyle. This method was supported by a campaign for screening dissemination. This methodological approach proved to be feasible, and the media campaign was successful at focusing on young and middle-aged adults, who could more likely suffer with sleep difficulties and excessive daytime sleepiness caused by rare hypersomnias.

## Data availability statement

The raw data supporting the conclusions of this article will be made available by the authors, without undue reservation.

## Ethics statement

The studies involving humans were approved by Ethical Commission of the Medical Faculty of University of Pavol Jozef Šafárik in Košice. The studies were conducted in accordance with the local legislation and institutional requirements. The participants provided their written informed consent to participate in this study.

## Author contributions

JH: Writing – original draft, Visualization, Software, Project administration, Methodology, Investigation, Data curation. AG: Writing – original draft, Supervision, Software, Project administration, Methodology, Formal analysis, Conceptualization. SC: Writing – review & editing, Project administration, Data curation. EF: Writing – review & editing, Supervision, Project administration, Conceptualization.
